# Establishment of a transdermal infection model with *Leishmania amazonensis*

**DOI:** 10.1186/s13071-025-07127-w

**Published:** 2025-12-22

**Authors:** Naiara Carla Manhães, Hozany Praxedes, Alisson Amaral Da-Rocha, Douglas Barroso de Almeida, Igor Bittencourt dos Santos, Elias Barbosa da Silva-Junior, Luciana Covre, Celio Geraldo Freire-de-Lima, Daniel Claudio Oliveira Gomes, Alda M. da-Cruz, Alessandra Marcia da Fonseca-Martins, Herbert Leonel de Matos Guedes

**Affiliations:** 1 Laboratório de Imunobiotecnologia, Instituto de Microbiologia Paulo de Gòes, Universidade Federal do Rio de Janeiro (UFRJ), Rio de Janeiro, RJ 21.941-902 Brazil; 2 Laboratório de Imunologia Clínica, Instituto Oswaldo Cruz, Fundação Oswaldo Cruz (Fiocruz), Rio de Janeiro, RJ 21.040-900 Brazil; 3 Laboratório de Imunomodulação, Instituto de Biofísica Carlos Chagas Filho (IBCCF), Rio de Janeiro, 21.941-902 RJ Brasil; 4 Núcleo de Doenças Infecciosas (NDI), Universidade Federal do Espírito Santo (UFES), Vitória, ES 29.043-900 Brazil; 5 Laboratório Interdisciplinar de Pesquisas Médicas (LIPMED), Instituto Oswaldo Cruz, Fundação Oswaldo Cruz (Fiocruz), Rio de Janeiro, 21.040-900 RJ Brasil; 6 Departamento de Microbiologia e Parasitologia (DMP), Universidade Federal do Estado do Rio de Janeiro (UNIRIO), Rio de Janeiro, RJ 20.211-040 Brazil; 7 Disciplina de Parasitologia, Faculdade de Ciências Médicas, Universidade do Estado do Rio de Janeiro, Rio de Janeiro, Brasil

**Keywords:** Leishmaniasis, Microneedle, Infection, Transdermal, Model, BALB/c, Ear

## Abstract

**Background:**

Leishmaniasis, a parasitic disease caused by *Leishmania* protozoa, has various clinical forms and is endemic in Brazil. Traditional experimental infection methods using intradermal and subcutaneous needles do not resemble natural sand fly transmission and are associated with risks of laboratory accidents owing to the use of low-gauge needles. In this study, we investigated the application of microneedles for transdermal (TD) infections to better replicate the deposition of parasites observed in the natural infection environment while ensuring safe handling in the laboratory.

**Methods:**

Initial experiments involved inducing TD infections using 1-, 7- or 12-microneedle cartridges, compared with inducing conventional intradermal (ID) infections in the ears of BALB/c mice. Subsequent tests used a 12-microneedle cartridge at different tissue depths (0.25, 0.5, 0.75, 1.0 and 1.5 mm), followed by challenge with different doses of *Leishmania amazonensis*. In addition, histology and flow cytometry studies were performed 30 min post-infection.

**Results:**

Transdermal infections produced similar lesion development and parasite loads as ID infections. More tissue destruction was induced using the 1- or 7-microneedle cartridge compared to the 12-microneedle cartridge and ID infection. Infection was successfully established at all microneedle depths (0.25, 0.5, 0.75 and 1.0 mm), resulting in comparable lesion thickness across the different experimental groups, with no statistically significant differences observed. The parasite load and lesion thickness were dependent on the inoculum size in the ear infection via the TD route, with efficient lesion progression using 2 × 10^6^ and 2 × 10^5^ promastigotes. Parasite loads in the ear were similar between the two approaches at the early stages, specifically at 30 min and 12 h post-infection. Thirty minutes post-infection, cytometry analysis indicated recruitment of neutrophils to the lesion site, regardless of the infection model used. The TD model specifically attracted neutrophils, independent of the presence of parasites.

**Conclusions:**

This novel microneedle infection model enables efficient challenge and reduces risks during animal experimentation. This technique holds promise for future studies of leishmaniasis therapy and vaccine development.

**Graphical Abstract:**

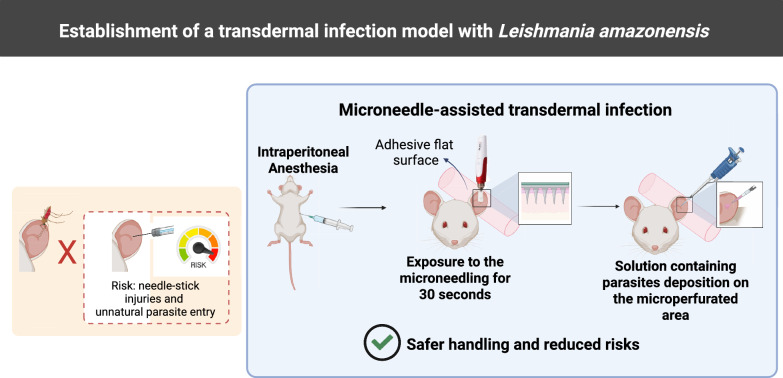

**Supplementary Information:**

The online version contains supplementary material available at 10.1186/s13071-025-07127-w.

## Background

Leishmaniasis is a neglected tropical disease that encompasses both cutaneous and visceral forms [1]. Cutaneous leishmaniasis, has multiple clinical forms and is the most common type of leishmaniasis, with between 600,000 and 1 million new cases estimated to occur worldwide each year [2]. In Brazil, the main causative agents of cutaneous leishmaniasis are *Leishmania* *amazonensis, Leishmania* (*Viannia*) *guyanensis* and *Leishmania* (*V*.) *braziliensis* [3]. *Leishmania amazonensis* causes a broad spectrum of diseases in humans, including cutaneous, diffuse, mucocutaneous and visceral leishmaniasis [4, 5], underscoring the importance of further research on *L. amazonensis* infection.

While administering substances in experimental in vivo models, several inoculation routes can be employed, each of which is intimately linked to the structure and function of different skin layers. Consequently, the choice of the inoculation route significantly influences the development of the immune response and absorption of the substance. For infections that involve parasites of the *Leishmania* genus, subcutaneous or intradermal (ID) routes are typically selected based on the parasite species, site of infection and study purpose [6]. Overall, for *Leishmania* infections, the subcutaneous route is preferred for paw infections, whereas the ID route is favored for ear infections.

Subcutaneous inoculation is a straightforward procedure [7–9]. In contrast, ID inoculation is considered to be more challenging because of the small gauge of the needle, tissue thickness and the risk of depositing the inoculum in an incorrect skin layer, as well as the higher likelihood of needle accidents [10]. However, based on sand fly infection, a model of infection closer to natural infection is preferable, with deposition of the parasite.

In this context, microneedles have been studied since 1976, and since 2000, there has been a significant increase in the number of publications utilizing these devices. This increase is due to the ability of the skin’s stratum corneum to mature and develop into the outer part of the epidermis, repair lesions and stimulate collagen production, among other factors [11–13]. Microneedles are manufactures in various types (e.g. solid, restored solid, dissolvable), materials (e.g. metals, silicon, polymers) and formats (e.g. derma-roller, derma-pen) [14]. Their use in therapy, diagnosis and aesthetic fields, such as in cancer and skin disease therapies, insulin administration, vaccines and biosensor construction, has been reported [13, 15].

Consequently, transdermal (TD) infection using microneedles has emerged as a promising alternative experimental in vivo model for infections. However, to date, there have been no reports describing the use of microneedles for this purpose. In the present study, a device equipped with solid metal microneedles was designed for TD delivery. This tool allows control over the speed and depth of microneedling as well as the number of microneedles arranged in the cartridge. Upon contact with the skin, microneedles create multiple punctures in the epidermis. Through these microchannel perforations, the inoculum can permeate the skin stratum and penetrate the skin layers. Therefore, our objective was to investigate the use of microneedles to establish an inoculation model of *L. amazonensis* in the ears of BALB/c mice, providing an alternative to the traditional method of ID infection.

## Methods

### Experimental animals

Isogenic female BALB/c mice, aged 6–8 weeks, were obtained from the Central Bioterium at the Centro de Ciências da Saúde, Universidade Federal do Rio de Janeiro, Brazil. These mice were housed in ventilated mini-isolators (Alesco) within the Experimental Bioterium at the Instituto de Microbiologia Paulo de Gòes (Rio de Janeiro). A clean, appropriate environment was maintained at a controlled temperature range of 20–25 ºC with regulated dark/light cycles (12/12 h); unrestricted access to water and food was provided. All animal experiments were conducted in accordance with the Brazilian Animal Protection Law (Lei Arouca, Nr. 11.794/08) and guidelines of the National Council for the Control of Animal Experimentation (CONCEA, Brazil). The experimental protocol was approved by the Committee for Animal Use of the Universidade Federal do Rio de Janeiro (UFRJ) (permit number A37/21-161-18). The randomization process and sample size calculation were based on the Cochran model [16]. Animals identified as statistical outliers were examined, but exclusion was not deemed necessary, as their presence did not have a significant effect on the overall results. The methods were in accordance with the Animal Research: Reporting of In Vivo Experiments (ARRIVE) guidelines.

### Parasite culture

*Leishmania amazonensis* (MHOM/BR/75/Josefa strain) promastigotes were used for in vivo infection. The Josefa strain was originally isolated from a human patient with cutaneous leishmaniasis [17]. The parasites were cultured at 26 °C in M199 medium (Difco Laboratories, Inc., Detroit, MI, USA) supplemented with 10% heat-inactivated fetal bovine serum (FBS; Cultilab, Guadalajara, Mexico) and 0.05% hemin (Sigma, St. Louis, MO, USA). Parasites were obtained from a limited dilution assay of macerated paws from infected mice. These parasites were maintained in culture until the fifth passage and used for experimental infection during the stationary phase.

### Animal anesthesia

Before in vivo infection, the animals were anesthetized with 130 μl of a solution containing a combination of xylazine and ketamine (Syntec, Taipei City, Taiwan, ROC) (4.3 mg/kg) administered intraperitoneally using a BD Ultra-Fine Insulin Syringe with a length of 6 mm and a gauge of 0.25 mm (BD, Franklin Lakes, NJ, USA).

### Experimental groups and in vivo infection

Infections were induced via both the TD and ID routes on the inner ear using *L. amazonensis* promastigotes in the stationary phase at concentrations of 2 × 10^6^, 2 × 10^5^, 2 × 10^4^ or 2 × 10^3^. TD infections were induced at varying needle depths (0.25, 0.5, 0.75, 1.0 and 1.5 mm), adjustable on the microneedling pen used (Dermapen®; MyM, Ultima N2-C, China) and equipped with disposable cartridges containing one, seven or 12 microneedles. The device was set to a microneedling speed of velocity 2, configured on the device itself. After the animal was anesthetized, the front part of the right external ear was adhered by a tape to a flat surface. The ears were then exposed to microneedling for 30 s. Following microneedling, a total volume of 10 µl containing the parasite solution was carefully distributed through the microperforated area using a pipette to ensure inoculum absorption. This TD method is shown in a video (Additional file 1: Video S1).

For ID infections, the same protocol for anesthetizing the mice was used, following which, the front part of the right external ear was adhered by tape to a flat surface, and a total volume of 10 µl containing the parasite solution was inoculated into the ear using a Mesoderm Alur® 34G and 8-mm slim needle (Alur Medical, Novo Hamburgo, Brazil).

Ear lesion thickness was measured using a Mitutoyo caliper (Mitutoyo Corp., Kawasaki, Japan). Initial measurements were obtained before infection, followed by weekly observations of lesion thickness. Lesion development was assessed by comparing the initial measurements with subsequent weekly measurements. Additionally, the macroscopic appearance of infected ears was documented using weekly photographs.

### Parasite load quantification

Parasite load was evaluated using a limiting dilution assay as previously described [16]. Briefly, the infected ears were excised and immersed in 70% alcohol for 1 min for disinfection. Subsequently, the spleen and draining auricular lymph nodes were excised. The organs were weighed and macerated in 1 ml M199 medium supplemented with 10% fetal bovine serum (FBS). For tissue analysis by flow cytometry, the ears were macerated in RPMI medium. A 96-well plate was prepared by adding 150 μl of M199 medium enriched with 10% FBS to each well. Next, 50 μl of the cell suspension was added to the first well. A 1:4 serial dilution process was performed, in which 50 μl of each dilution was transferred into the subsequent well, resulting in 24 dilutions for each sample. The plates were then stored at 26 °C in a bio-oxygen demand (BOD) incubator for 7–14 days. After incubation, the last well (last dilution) showing promastigote growth, as determined by visual inspection under a light microscope, was identified. This well was used as a reference point to calculate the total parasite count within an organ (parasite load). The amount of the parasite was then divided by tissue weight in grams (parasite load/tissue gram).

### Isolation of dermal cells from mice ears

After the parasite load had been quantified, the macerated ears were centrifuged at 400 *g* for 5 min at 4 °C to collect the supernatant. The tissues were then incubated for 30 min with 500 µl collagenase I and II (Sigma; 100 ug/ml) and RPMI (Sigma) solution at 37 °C. The control experiment was performed using ears from naïve mice. Following incubation, the tissue was macerated with a tissue mixer for 3 min, filtered through a 70-µm cell strainer and washed with 4 ml of phosphate buffered saline (PBS) at 400 g for 5 min at 4 °C. The cells were collected and resuspended in RPMI/10% FBS for further use.

### Cell staining for flow cytometry

The ear cells were washed with PBS by centrifugation at 400 *g* for 5 min at 4 °C, blocked with 5 µl/well anti-Mo CD16/CD32 (clone 93) (eBioscience, Carlsbad, CA, USA) (1:50) for 15 min, followed by the addition of 5 µl/well of an antibody cocktail for extracellular staining: anti-CD45 PB (clone 30-F11; BioLegend, San Diego, CA, USA; 1:100), anti-Ly6g BV510 (clone 1A8; BioLegend; 1:100) and anti-CD11b PE Cy7 (clone M1/70; BioLegend; 1:100), with incubation for 30 min at 4 °C in the dark. Finally, the cells were washed with a cytometry buffer solution (PBS with 5% FBS), resuspended in cytometry buffer solution and stored in the dark at 4 °C until acquisition. Analysis was performed using FlowJo software vX.0.7 (BD) and graphics were generated using GraphPad Prism 8.0.2 (GraphPad Software, San Diego, CA, USA).

### Histological analysis

BALB/c mouse ears were transdermally or intradermally infected with 2 × 10^6^ *L. amazonensis* inoculum and excised with a scalpel 30 min later. The ears were fixed in formalin and then embedded in paraffin. The fixed samples were sent to the Histotechnology Platform on FIOCRUZ/RJ for paraffin processing, microtomy (slice thickness: 5–6 µm) and staining with hematoxylin and eosin (H&E). Lamin analysis and photographs were captured with a digital slide scanner 3DHISTECH Panoramic 1000X (3DHISTECH Ltd. Budapest, Hungary).

### Data analysis

Prior to conducting the experiments, a statistical power analysis was performed using G*Power software to determine the appropriate number of animals per group and ensure sufficient statistical sensitivity to detect biologically relevant differences. We used BALB/c mice, a strain whose data typically follow a parametric distribution. Nevertheless, we conducted formal tests of normality for all analyzed variables, including the Shapiro–Wilk test, visual inspection via Q–Q plots and comparison of mean, median, skewness and kurtosis values. These analyses confirmed that the data met the assumptions for the use of parametric tests. For comparisons between two experimental conditions, we applied an unpaired t-test. For experiments involving ≥ 3 groups, we employed one-way analysis of variance (ANOVA) followed by appropriate post hoc tests for multiple comparisons. In the specific case of lesion progression over time, we used two-way ANOVA, as this analysis involves two independent variables (time and treatment)—a well-established approach in the literature for this type of experimental design. Data analysis was performed using GraphPad Prism software (version 8.0; GraphPad Software). The statistical significance of the differences between the mouse groups was determined using either a two-way ANOVA or Student's t-test. The values are presented as the mean ± standard deviation (SD) (Statistical significance was set at p≤ 0.5).

## Results

### Lesion thickness and parasite load were similar in TD-induced infection and ID-induced infection

To assess the efficacy of our TD ear infection model and to determine the optimal number of microneedles required, we infected BALB/c mice with 2 × 10^6^ *L. amazonensis* promastigotes. The animals were transdermally infected using a 1.0-mm depth configuration with cartridges containing 12 microneedles (TD12), seven microneedles (TD7) or one microneedle (TD1), or by the ID method.

**Fig. 1:**
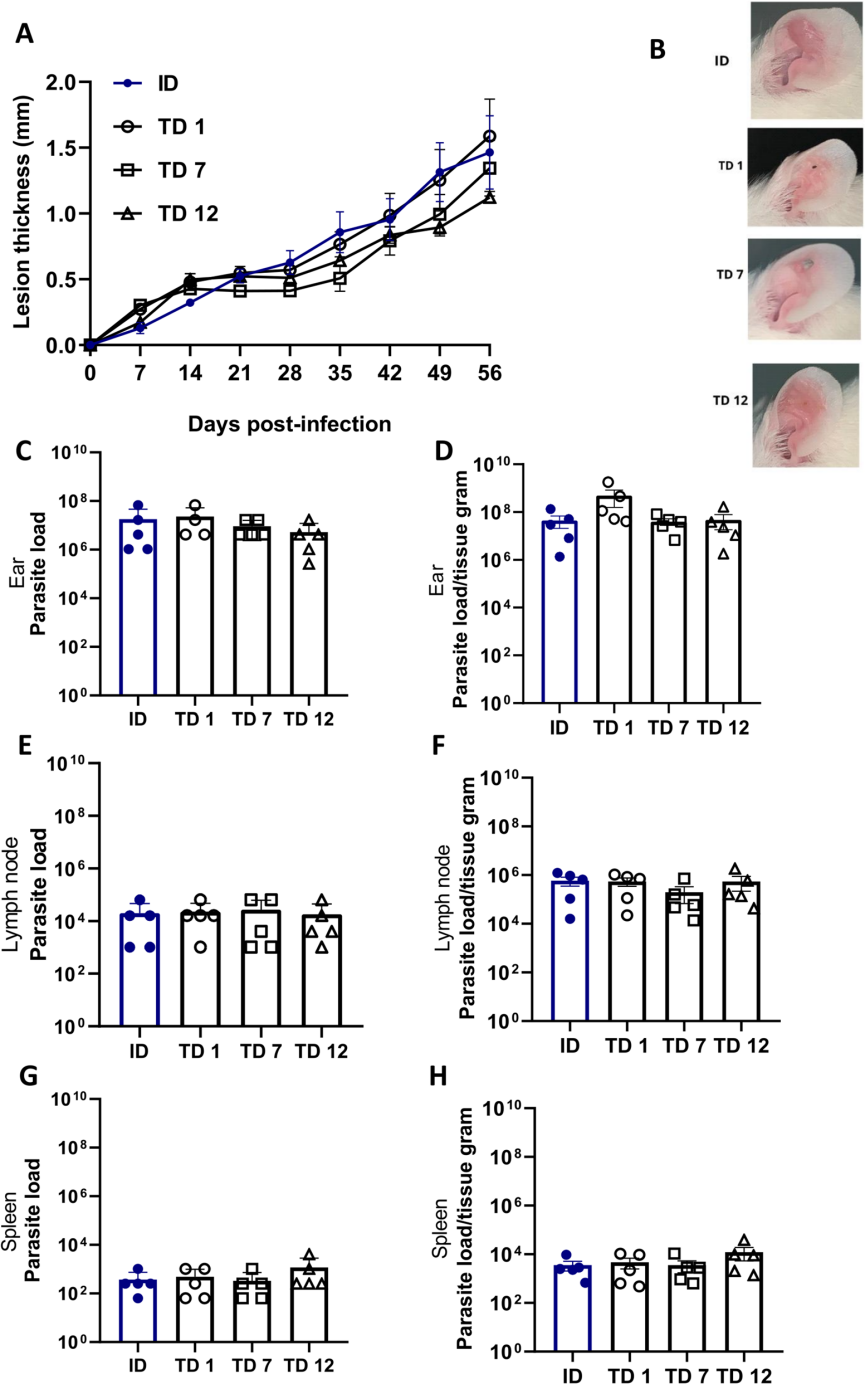
Transdermal ear infection in BALB/c mice induces lesions characterized by lesion thickness and parasite load similar to those associated with ID ear infection. Female BALB/c mice aged 6–8 weeks were infected with 2 × 10^6^ *Leishmania* *amazonensis* promastigotes in the stationary phase, either through the TD route using cartridges with different quantities of microneedles (12, 7 and 1) or by the ID route. Ear lesion thickness and macroscopic aspect were assessed over 56 days, after which the animals were euthanized for the limited dilution assay. **A** Lesion thickness (mm) per day post-infection, **B** representative ear lesion photographs. **C**, **E**, **G** Quantification of parasite load of the ear (**C**), draining lymph node (**E**) and spleen (**G**). **D**, **F**, **H** Quantification of parasite load per gram of tissue of the ear (**D**), draining lymph node (**F**) and spleen (**H**). Data are presented as the mean  ± standard deviation (*n* = 5) and are representative of three independent experiments producing the same result profile. ID, Intradermal; TD, transdermal; TD1, TD7, TD12, number of microneedles in the cartridge used in the TD infection procedure

The lesion thickness induced in the TD infection model was similar to that produced using the ID method, with no significant differences observed between the 7-microneedle and 12-microneedle cartridges (Fig. 1A). The 12-microneedle configuration alone did not result in tissue destruction, as revealed by the ear photographs (Fig. 1B; Additional file 2: Figure S1). Parasite quantification showed that lesions formed in the TD model, regardless of needle configuration, contained parasite quantities similar to those found in the ID model (Fig. 1C, D). This pattern was consistent in the draining lymph nodes (Fig. 1E, F) and spleens (Fig. 1G, H), with no significant differences between the ID and TD groups.

These data highlight the promising potential of the TD infection model, as evidenced by comparable parasite loads in the draining lymph node, ear and spleen compared to mice subjected to ID infection with an equivalent inoculum, regardless of the number of microneedles in the cartridge. Also, based on these results, the 12-microneedle cartridge was established as a parameter for our TD model, as its use not only induced lesion and parasite loads comparable to those of the other groups but also avoided tissue destruction at the infection site.

### Optimization of the TD infection model using 1.0-mm-deep microneedles

**Fig. 2:**
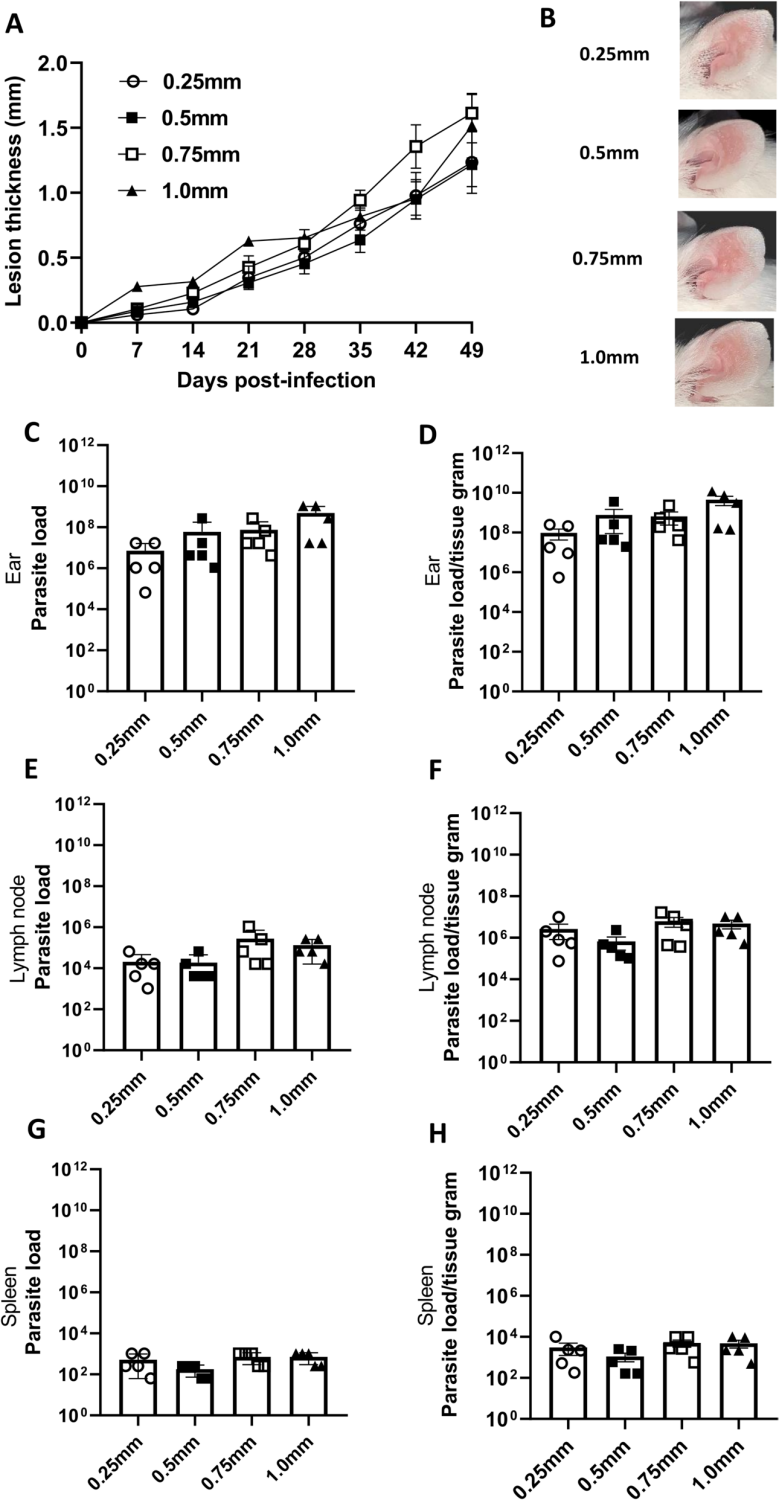
Optimization of the TD ear infection model using *Leishmania* *amazonensis* was achieved by employing microneedles set at a depth of 1.0 mm. Female BALB/c mice aged 6–8 weeks were infected with 2 × 10^6^ *L*. *amazonensis* promastigotes in the stationary phase, either by the TD route at different microneedle depths (from 0.25 to 1.0 mm) or by the ID route. Mice lesion thickness and macroscopic aspect were assessed over 49 days, after which the animals were euthanized for the limited dilution assay. **A** Lesion thickness (mm) per day post-infection. **B** Representative ear lesion photographs. **C**, **E**, **G** Quantification of parasite load of the ear (**C**), draining lymph node (**E**) and spleen (**G**). **D**, **F**, **H** Quantification of parasite load per gram of tissue of the ear (**D**), draining lymph node (**F**) and spleen (**H**). Data are presented at the mean ± standard deviation (*n* = 5) and are representative of three independent experiments producing the same result profile. ID, Intradermal; TD, transdermal

We also explored another critical parameter essential for establishing a TD ear infection model: the depth reached by the microneedles into the tissue. Ears of BALB/c mice were exposed to 2 × 10^6^ *L. amazonensis* promastigotes using microneedles at different tissue depths. The resulting lesions were evaluated over a period of 49 days using a combination of ear thickness measurements and weekly photographic documentation (Additional file 3: Figure S2). To determine the appropriate microneedle depth for TD parasite delivery, we initially tested both 1.5-mm and 1.0-mm microneedles. However, the 1.5-mm microneedles frequently penetrated completely through the ear tissue, resulting in leakage of the inoculum and loss of control over the infection site. In contrast, the 1.0-mm microneedles provided sufficient tissue resistance to retain the inoculum and allowed for more consistent delivery (Additional file 4: Figure S3; Additional file 5: Figure S4). These findings led us to investigate shorter microneedles (0.25, 0.5 and 0.75 mm) in subsequent experiments to refine the method and minimize variability (Fig. 2). Notably, infection was successfully established at all microneedle depths (Fig. 2), resulting in comparable lesion thickness across the different groups, with no statistically significant differences observed (Fig. 2A).

Analysis of ear photographs showed lesion progression across all experimental groups (Fig. 2B; Additional file 3: Figure S2). Analysis of parasite load in all groups infected via the TD model showed a consistent parasite load independent of the depth of the microneedle (Fig. 2C–H). Thus, the 1.0-mm configuration was chosen as it displayed a similar lesion and parasite load to those of the other configurations available, and was assessed as being suitable for the thickness of the infection site.

### Parasite load and lesion thickness were dependent on the inoculum size in the ear infection via the TD route

After establishing key parameters of microneedle usage in our model, we aimed to evaluate the impact of varying quantities of parasites on TD ear infection. To assess this effect, BALB/c mice were divided into four groups, each infected with distinct quantities of *Leishmania*, with the first group receiving 2 × 10^6^ parasites; the second group, 2 × 10^5^ parasites; the third group, 2 × 10^4^ parasites; and the fourth group, 2 × 10^3^ parasites.

**Fig. 3:**
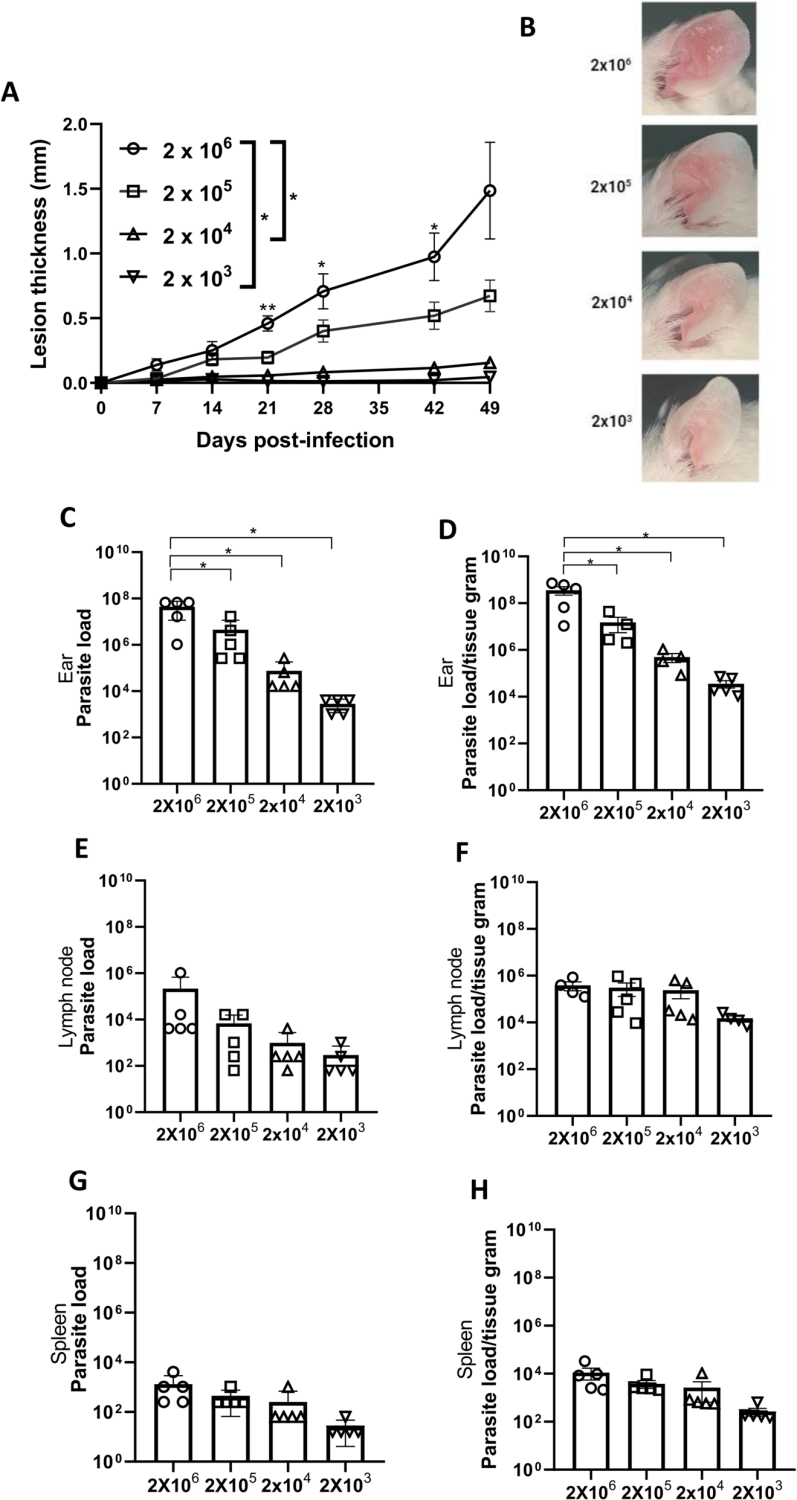
Parasite load and lesion thickness were inoculum dose-dependent in the ear infection by the transdermal route. Female BALB/c mice aged 6–8 weeks were infected with different parasite quantities of *Leishmania* *amazonensis* promastigotes in the stationary phase (2 × 10^6^, 2 × 10^5^, 2 × 10^4^ and 2 × 10^3^) by the TD route employing a 12-micro needle cartridge at a depth of 1.0 mm. Ear lesion thickness and macroscopic aspect were assessed over 49 days, after which the animals were euthanized for limited dilution assay. **A** Lesion thickness (mm) per day of infection. **B** Representative ear lesion photographs. **C**, **E**, **G** Quantification of parasite load of the ear (**C**), draining lymph node (**E**) and spleen (**G**). **D**, **F**, **H** Quantification of parasite load per gram of tissue of the ear (**D**), draining lymph node (**F**) and spleen (**H**). Data are presented at the mean ± standard deviation (*n* = 5) and are representative of three independent experiments producing the same result profile. Asterisk indicates a significant difference at *p ≤ 0.05 compared to 2 × 10^6^ promastigotes

The results showed that the groups infected with 2 × 10^6^ and 2 × 10^5^ parasites exhibited greater lesion growth than those infected with the lower doses (Fig. 3A, B). However, all groups presented significant parasite load in the ear (Fig. 3c, d), draining lymph nodes (Fig. 3E, F) and spleen (Fig. 3G, H), but particularly in the ear (Fig. 3C, D). In the comparative analysis, all animals exhibited redness at the infection site, with the group infected with 2 × 10^6^ parasites showing the most pronounced infection intensity (Fig. 3B).

This result suggests that although this method induces a dose-dependent response in the ear, even small inoculum doses can result in detectable parasite loads and promote visceralization, independently of the inoculum dose and lesion development.

### Evaluation of early-infection aspects in the TD model

**Fig. 4:**
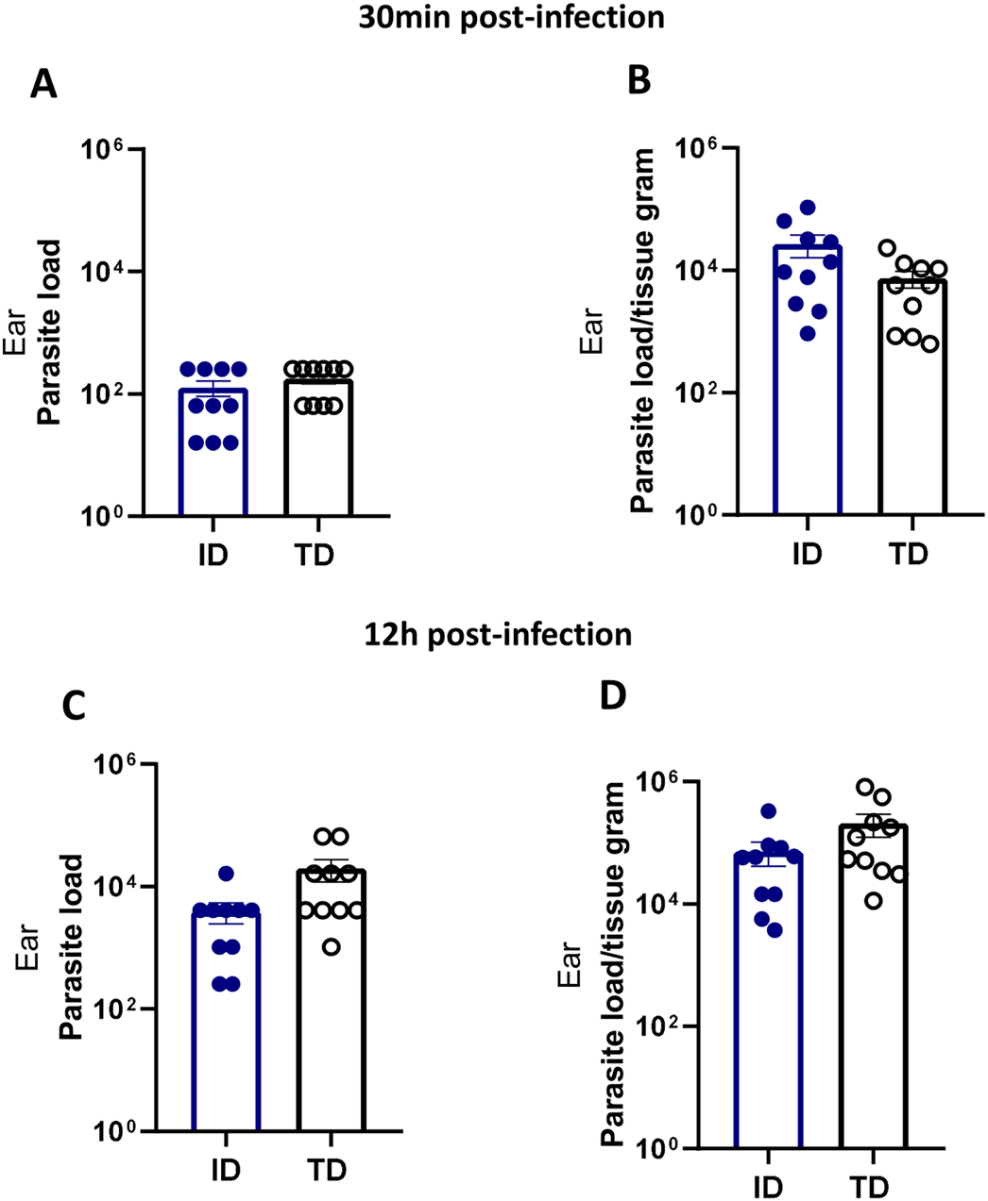
Parasite load at early-infection timepoints. Female BALB/c mice aged 6–8 weeks were infected with 2 × 10^6^* Leishmania* *amazonensis* promastigotes by the TD route using a 12-microneedle cartridge at a tissue depth of 1.0 mm or by the ID route. Animals were euthanized for the limited dilution assay at 30 min and 12 h after the infection. Quantification of total parasite load. Quantification of parasite load per gram of tissue. The data are presented at the mean ± standard deviation (*n* = 5) and are representative of two independent experiments producing the same result profile. ID, Intradermal; TD, transdermal

Another stage of the study aimed to determine the early detection of parasite presence following infection and to determine the number of parasites that need to be used to achieve infection. BALB/c mice were intradermally inoculated with 2 × 10^6^ *L. amazonensis* parasites in the ear and transdermally inoculated using a cartridge with 12 microneedles. Parasite load in the ear was assessed at 30 min and 12 h post-infection. The results indicated that parasites were present in the ear lesions at both timepoints, irrespective of the route of infection. Both the ID and TD infection routes showed similar parasite loads (Fig. 4).

These findings suggest that despite the differences in the infection method, the parasite loads in the ear were comparable between the two infection approaches at the early infection timepoints of 30 min (Fig. 4A, B) and 12 h post-infection (Fig. 4C, D).

**Fig. 5:**
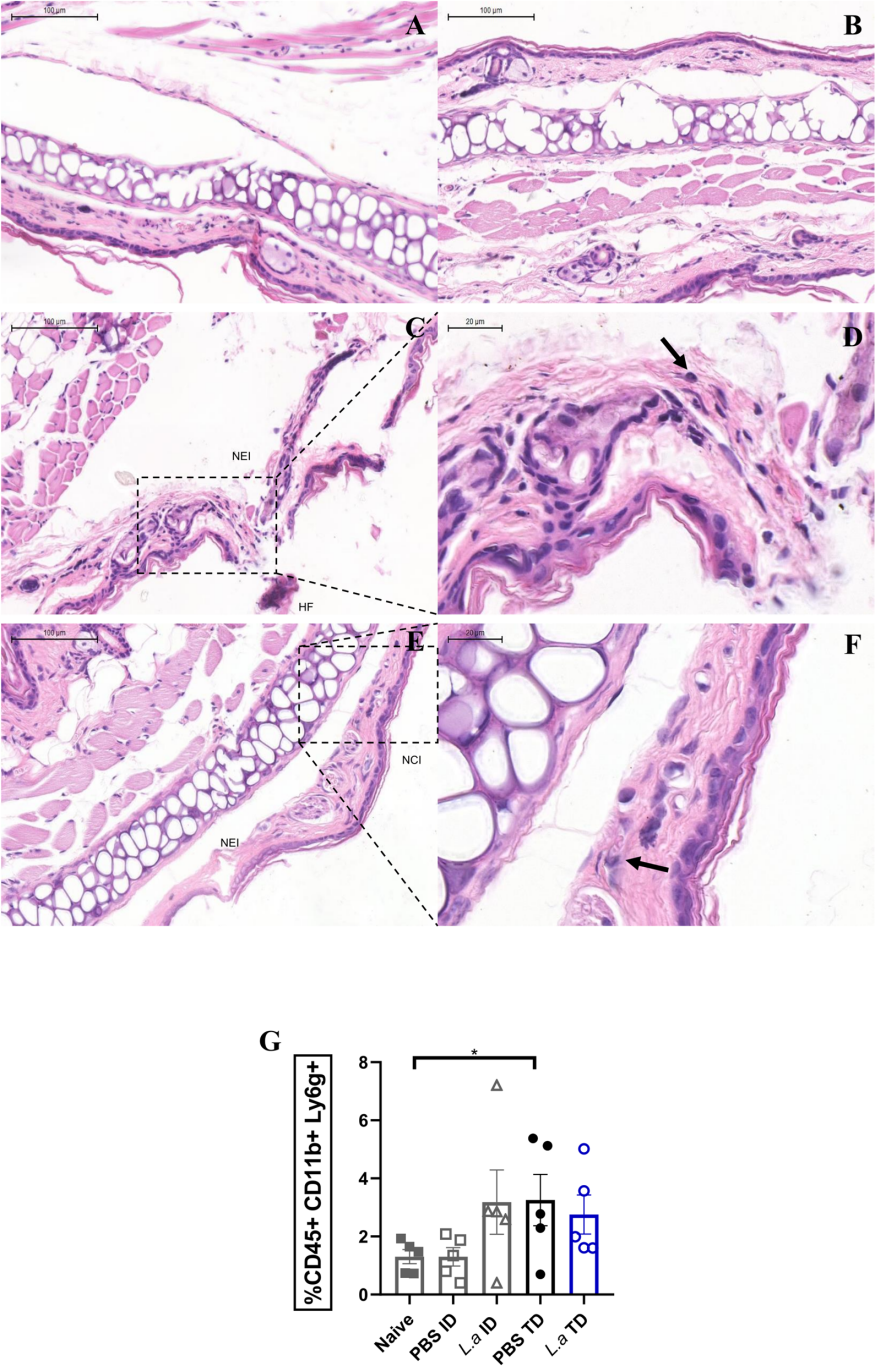
Parasites could be seen early in the TD infection process accompanied by neutrophil recruitment. Female BALB/c mice aged 6–8 weeks were infected with 2 × 10^6^ *Leishmania* *amazonensis* promastigotes by the TD route using a 12-needle cartridge at a tissue depth of 1.0 mm or by the ID route. Mice were euthanized and the ears were collected for histological analysis 30 min after the procedure as described in the Methods section. Histological sections of ears treated with PBS using the ID (**A**) or TD method (**B**).** C**–**F** Histological sections of ears infected with *L. amazonensis* by the ID (**C**, **D**) or TD method (**E**, **F**). Black arrows indicate neutrophils. The histological sections were stained with hematoxylin and eosin. **G** Neutrophil frequency in the ear tissue after both methods, with and without the parasites. Data are presented as the mean ± standard deviation (*n* = 5) and are representative of two independent experiments producing the same result profile. HF, Hemorrhagic focus; ID, Intradermal; L.a, * Leishmania* *amazonensis*; NCI, needle-induced cartilage Injury; NEI, needle-induced epithelial injury; PBS, phosphate buffered saline; TD, transdermal

Ear tissue was histologically analyzed at 30 min after infection with *L. amazonensis* to characterize immunological cell infiltration (Fig. 5). In the control groups, ears injected with PBS served as references for the procedures. The ID control group (Fig. 5A) displayed preserved tissue architecture with intact dermal and epidermal layers, whereas in TD control group (Fig. 5B) had discrete punctures traversing the epidermal layer, consistent with the microneedling process and without additional tissue disruption.

As a result of needle puncture, morphological alterations are observed in the ears from infected mice after ID, such as dermal disruption, lacerations and fibrillar fragmentation caused by needle-induced epithelial injury (NEI) (Fig. 5C, D). On the other hand, NEI and needle-induced cartilage injury (NCI) are less pronounced in the ears of infected mice after the TD procedure (Fig. 5F, G).

The cellular infiltrate in both models indicates the presence of polymorphonuclear cells with a slightly eosinophilic cytoplasm, observed in the connective tissue after 30 min, indicating the presence of neutrophils (Fig. 5D, F). However, the cellular infiltrate is poor in lymphocytes and other non-resident cells, as expected. At the same time point, neutrophil infiltration was observed in the ear tissue after both infection methods using flow cytometry (Fig. 5G). Our observations indicate that there is an equivalent recruitment between the methods, with approximately 3% of neutrophils in the TD method, both with parasites and PBS, and in the ID method. In addition, Neutrophils can be recruited using the TD method independently of parasite presence, at almost twofold more often as with the ID method with PBS medium only.

## Discussion

The ID administration method faces challenges in precisely targeting the appropriate skin layer, and these often result in the inoculum being misdirected to an unintended skin stratum or even penetrating beyond the skin layers, leading to inoculum loss [9, 10]. In mice, this issue is compounded by the thinness of the mouse external ear (250–300 µm on average) combined with the need to utilize low-gauge needles, which increases the potential for errors [17]. TD administration is performed at a 90 ° angle to the mouse ear, while in the ID method, the needle is angled at 10–15 ° [18–20]. Taken together, the TD route offers a significant advantage by reducing these errors during administration, thereby enhancing operator safety.

Macroscopic differences were observed between lesions established using the TD and ID models. In the ID infection group, a single edema formed at the infection site, whereas the TD infection groups exhibited multiple centers of edema due to repeated puncturing by the microneedle apparatus. The latter resembles natural infection, as phlebotomine sand flies are known to repeatedly puncture the host's skin to access the bloodstream while feeding, even when phlebotomine sand flies fail to fully feed or do so only partially, infection can still develop [21, 22]. This repeated puncturing behavior results in multiple lesions, similar to those created by the TD infection model.

The 7-microneedle and 1-microneedle cartridge configurations caused tissue destruction, whereas the 12-microneedle cartridge caused significant lesions without extensive tissue loss. The microneedles in the 7- and 1-microneedle cartridges are centrally located, whereas those in the 12-microneedle cartridge are distributed equidistantly in a circular pattern. The pressure equation, which states that the pressure equals the force divided by the perpendicular area over which it is applied [23], therefore explains this result. The impact of the force applied by the device varied significantly, depending on the distribution area.

The depth of penetration of the different sand fly species that can transmit leishmaniasis corresponds to the length of the labrum, a part of the sand fly’s mouth apparatus [24]. This variation in labrum length affects the depth to which each sand fly species can penetrate the skin, suggesting that sand flies may not always reach blood vessels, depending on their labrum length [25]. In the TD model, ear lesions with *L. amazonensis* were established at depths of 0.25, 0.5, 0.75, 1.0 and 1.5 mm, with all of these lesions showing comparable macroscopic appearance, thickness and parasite loads, including in the spleen. This depth range is consistent with the thickness range that sand flies are capable of reaching.

Studies quantifying the number of promastigotes ejected by infected sand flies using artificial feeding systems have found that the inoculum size can range from 10 to nearly 100,000 *Leishmania* cells [26]. In establishing experimental infections with *L. amazonensis*, many researchers have used inocula containing high quantities of parasites to ensure successful infection establishment. However, this approach diverges from the natural dynamics of infection. In their study employing the ID method to infect BALB/c mouse paws with *L. major* [27], Courret et al. reported that fewer promastigotes resulted in the absence of visible or progressive lesions. However, these authors did note that some of the animals exhibited enlarged draining lymph nodes with detectable parasites, which is consistent with our findings. In contrast, Felizardo et al. [28], using C57BL/6 mice, observed a lower parasite load, but also the development of visible lesions despite a delay in their development. These differences may be due to differences in the immune response of these animal strains, indicating that BALB/c mice, even those susceptible to *L. major*, can control parasite proliferation when facing challenges with low parasite doses [29].

The same may have occurred in the present study, with *L. amazonensis*, as BALB/c mice have also been shown to present a susceptibility profile to this* Leishmania* species [30, 31]. In the present study, we observed that higher inoculum doses resulted in larger lesions. However, mice infected with lower doses did exhibit detectable parasite loads in the ear, draining lymph nodes and spleen. Notably, parasites were found in the spleens of animals infected transdermally with a 1-mm microneedle, even at lower inoculum doses. This corresponds with findings reported previously in *Leishmania mexicana*, a species that is from the same complex as *L*. *amazonensis*, which demonstrated systemic dissemination and parasitemia in the spleens of BALB/c mice at low doses [32].

The host immune response plays a critical role in the outcome of *Leishmania* infection, which varies among species. For example, during ID infections, immune cells such as neutrophils and dendritic cells are recruited and activated at the infection site [33–35]. This recruitment and activation during ID injections has been shown to be facilitated by increased local pressure in the interstitial tissue caused by the needle, which enhances capillary permeability and subsequently increases lymphatic flow [36]. Both the TD and ID infection methods recruit neutrophils to the infection site, but the TD method recruits neutrophils in the presence or absence of parasites (Fig. 5I), indicating that the method itself is capable of establishing a proper environment for the parasite to establish [37, 38]. In addition, histological sections showed neutrophils in the needle injury area caused by both ID and TD administration after 30 min (Fig. 5D, F). Although the cellular infiltrate in the needle injury area is modest and poor in non-resident immune cells, neutrophils are present 30 min hour after *Leishmania* inoculation by both the ID and TD routes (Fig. 5D, F). The recruitment was also observed by flow cytometry (Fig. 5G). The increase in CD45^+^CD11b^+^Ly6G^+^ cells was observed in both the ID and TD infection groups inoculated with *L*. *amazonensis*. Interestingly, neutrophils were also detected in the TD group that received only PBS, suggesting that this method can provide novel insights by recapitulating key features of cutaneous leishmaniasis. This response may result from the release of DAMPs that recruit neutrophils, analogous to the inflammatory response induced by a sand fly bite, which occurs independently of the presence of *Leishmania* spp. [39].

In the TD method, the parasite is deposited, whereas in the ID method, the parasite is inoculated. This highlights the relevance of the TD approach as a robust and consistent method for studying the early events of *Leishmania* infection in the skin.

One potential limitation of the TD infection approach is the inability to quantify the exact number of parasites that effectively penetrate the tissue at the time of application. While a fixed inoculum volume containing a known concentration of parasites was applied to all animals, and microneedle disruption was standardized, individual variation in skin permeability could lead to differences in initial parasite uptake. However, the limited dilution assay performed 30 min and 12 h post-application in our study revealed the presence of parasites in the ear tissue, supporting the effectiveness and reproducibility of the method. Still, we recognize that this aspect may introduce a degree of variability, which should be considered in future refinements of the model.

This study benefits researchers and academics working in laboratories whose activities require performing *Leishmania* sp. in vivo infections. People with anxiety sensations associated with needles and injections have been described and, according to the* Diagnostic and Statistical Manual of Mental Disorders*, fifth edition (DSM-5), people who have this condition are susceptible to stimuli that make them avoid visual contact with the object, followed by low blood pressure, which can result in a fainting episode [40]. According to a meta-analysis by McLenon and Rogers [41], aichmophobia (i.e. an irrational fear of sharp objects) is a known phobia and is more prevalent in children up to 5 years of age, with a prevalence ranging from 20% to 30% in adults aged 20 to 40 years. Therefore, it is probable that there are individuals in the academic/research environment with this phobia who are unable to perform needle manipulation and injections. The microneedles used in this study were partially occulted into the cartridge that was coupled to the device; as such no visible signs of injection were visible, which may diminish the visual stimuli that could trigger aichmophobia symptoms.

As previously observed, compared to the TD method, the ID method intrinsically provides more difficult access to the correct layer of the skin, which increases the chances of the inoculum going to another (incorrect) stratum. Additionally, the mouse’s external ear is a structure of low thickness, which can multiply the risk factors during ID injections. Therefore, another benefit of the microneedles is the lowered chance of errors during inoculation because besides to accessing the ear at 90 °, the inoculum is placed after the perforation in the ear has been made.

It is essential to acknowledge that the TD method employed in this study markedly differs from both natural sand fly transmission and conventional needle inoculation. Unlike natural vector-borne infection, which involves the co-inoculation of *Leishmania* parasites with sand fly saliva, promastigote secretory gel (PSG) and commensal microbiota—all of which modulate the host immune response [42–44]—the microneedle approach bypasses these critical elements. Instead, parasites are deposited externally, in contact with air, and may enter tissue passively through microneedle-induced damage.

The skin microbiota is also a relevant factor, as it plays a role in natural vector-mediated infection, needle inoculation [33, 45, 46] and likely in our TD model as well. Notably, the TD method may further amplify the influence of the microbiota due to the greater extent of tissue disruption caused by the 12-microneedle configuration—an effect that resembles the mechanical damage induced by sand fly bites during infection attempts. However, the exact contribution of the skin microbiota in our model remains to be determined and requires further investigation.

While this model does not aim to replicate natural transmission, it provides a distinct, safe and useful platform to explore immune responses, therapies and vaccines in *Leishmania* experimental infections.

## Conclusions

In this study, we introduced a novel model for performing in vivo ear infections in the laboratory. Utilizing the transdermal route, we demonstrated that this infection method can induce lesions and parasite loads comparable to the traditional intradermal method in both early and chronic stages of infection. This innovative approach ensures the safety and effectiveness of the infection.

## Supplementary Information

**Additional file 1: Video S1. **Transdermal infection demonstration. Video demonstration of the transdermal infection model in BALB/c mouse ears.

**Additional file 2: Figure S1.** Weekly ear photographs of animals infected with different quantities of microneedles. (A) Animals infected by the intradermal route (ID); (B) Animals infected with 1-needle cartridge; (C) Animals infected with 7-needle cartridge; (D) Animals infected with 12-needle cartridge. The data (means ± standard deviations; *n* =5) are representative of three independent experiments producing the same result profile.

**Additional file 3: Figure S2.** Weekly ear photographs of animals infected with different depths of microneedling. (A) Animals infected using 0.25mm microneedling depth; (B) Animals infected using 0.5mm microneedling depth; (C) Animals infected using 0.75mm microneedling depth; (D) Animals infected using 1.0mm microneedling depth. The data (means ± standard deviations; *n* =5) are representative of three independent experiments producing the same result profile.

**Additional file 4: Figure S3. **Configuration of 1.5-mm depth is not optimal for the transdermal model of infection. Female BALB/c mice aged 6-8 weeks were infected with 2x10^6^ *Leishmania amazonensis *promastigotes in the stationary phase, either by transdermal route (TD) with different microneedle depth (1.5mm and 1.0mm). Mice lesion thickness and macroscopic aspect were assessed over 49 days, after which the animals were euthanized for Limited dilution assay. (A) Lesion thickness in millimeters per days of infection; (B) Representative ear lesion photographs; (C) Quantification of parasite load per tissue gram of the ear; (D) and draining lymph node. The data (means ± standard deviations; *n* = 5) are representative of two independent experiments producing the same result profile.

**Additional file 5: Figure S4.** Configuration of 1.5-mm depth caused tissue destruction. Female BALB/c mice aged 6-8 weeks were infected with 2x10^6^ *Leishmania amazonensis *promastigotes in the stationary phase, either by transdermal route (TD) with different microneedle depth (1.5mm and 1.0mm). Weekly photographs of mice lesion macroscopic aspect was assessed over 49 days. The data (means ± standard deviations; *n* = 5) are representative of two independent experiments producing the same result profile.

## Data Availability

All data is available in the manuscript.

## Abbreviations

ID: Intradermal
TD: Transdermal
