# Supplementary figures and images for: Establishment of a transdermal infection model with Leishmania amazonensis
Source: Parasit Vectors. 2025 Dec 22;19:74. doi: 10.1186/s13071-025-07127-w (PMC12882241; doi:10.1186/s13071-025-07127-w)

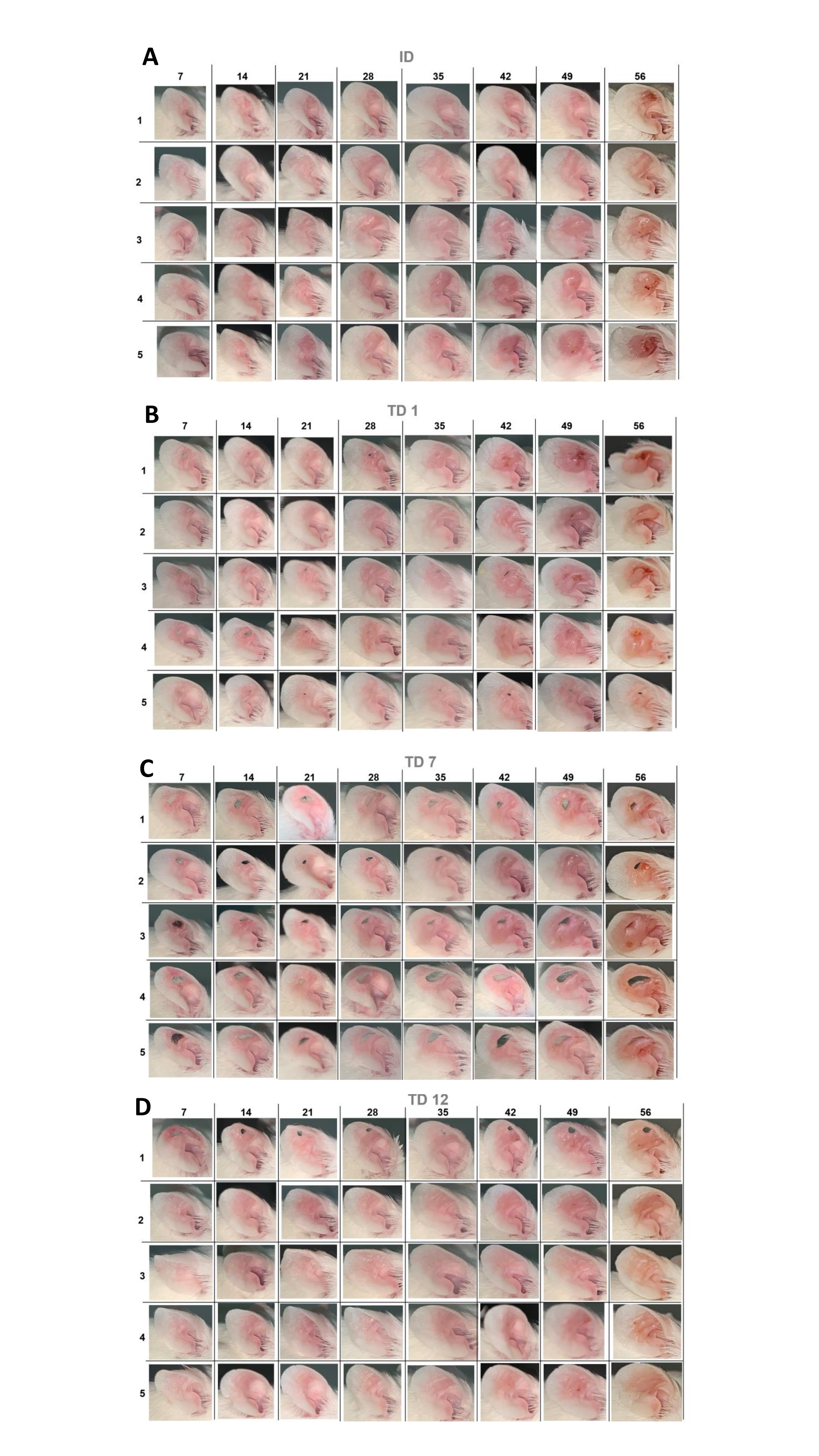

Supplement: Supplementary file 2 — Additional file 2: Figure S1. Weekly ear photographs of animals infected with different quantities of microneedles. (A) Animals infected by the intradermal route (ID); (B) Animals infected with 1-needle cartridge; (C) Animals infected with 7-needle cartridge; (D) Animals infected with 12-needle cartridge. The data (means ± standard deviations; n =5) are representative of three independent experiments producing the same result profile. [file 13071_2025_7127_MOESM2_ESM.tif]

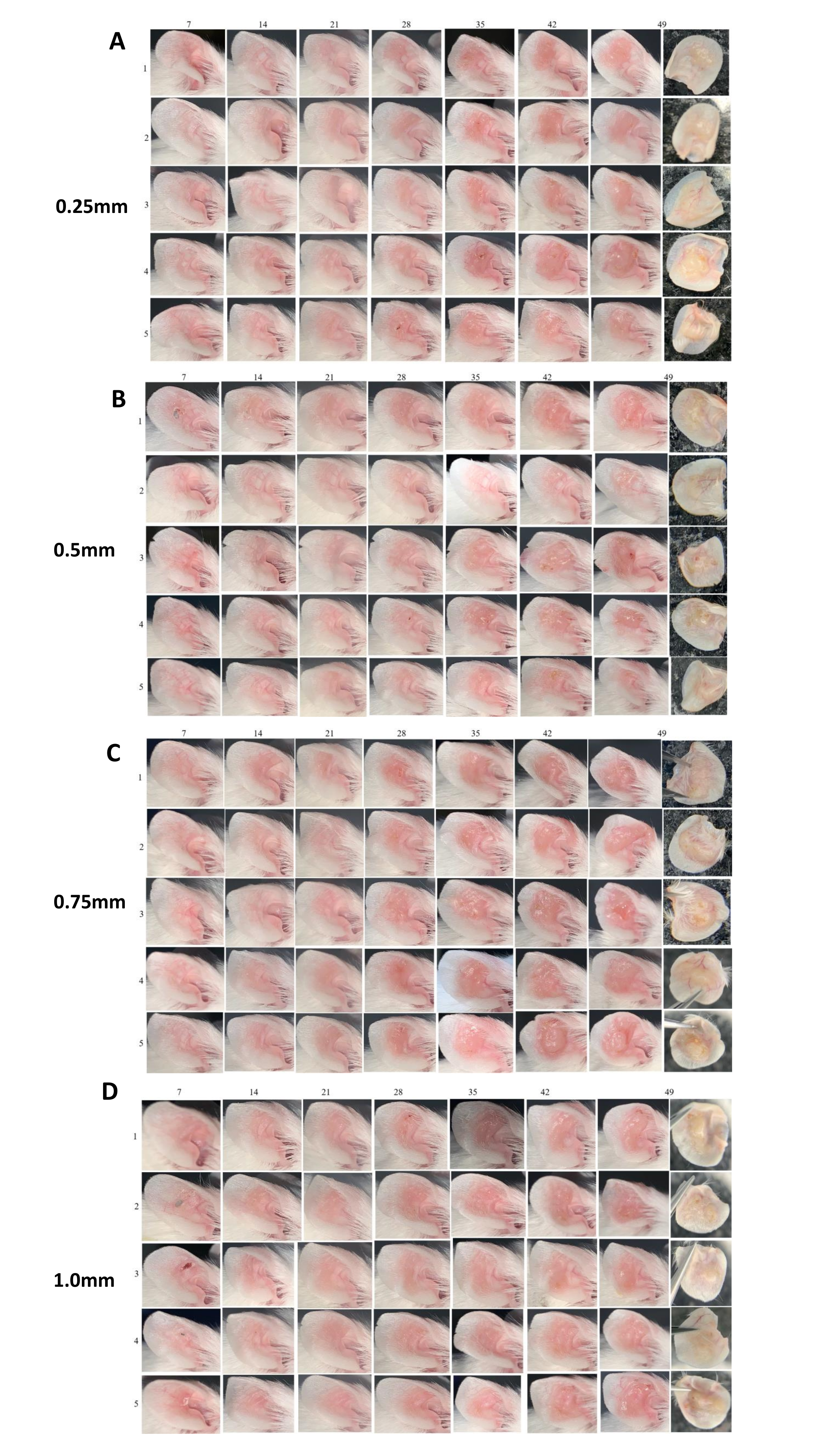

Supplement: Supplementary file 3 — Additional file 3: Figure S2. Weekly ear photographs of animals infected with different depths of microneedling. (A) Animals infected using 0.25mm microneedling depth; (B) Animals infected using 0.5mm microneedling depth; (C) Animals infected using 0.75mm microneedling depth; (D) Animals infected using 1.0mm microneedling depth. The data (means ± standard deviations; n =5) are representative of three independent experiments producing the same result profile. [file 13071_2025_7127_MOESM3_ESM.tif]

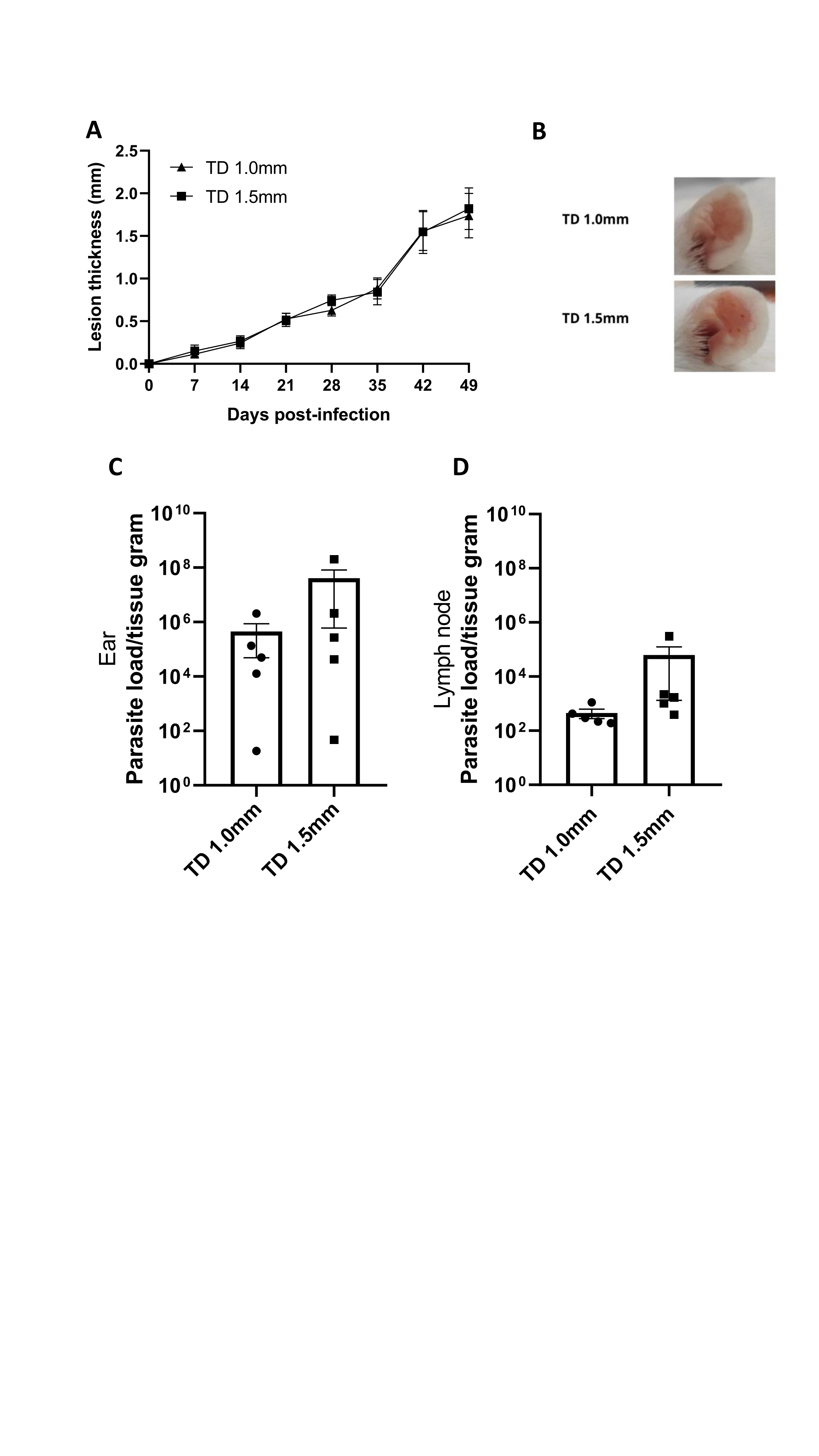

Supplement: Supplementary file 4 — Additional file 4: Figure S3. Configuration of 1.5-mm depth is not optimal for the transdermal model of infection. Female BALB/c mice aged 6-8 weeks were infected with 2x10^6^ Leishmania amazonensis promastigotes in the stationary phase, either by transdermal route (TD) with different microneedle depth (1.5mm and 1.0mm). Mice lesion thickness and macroscopic aspect were assessed over 49 days, after which the animals were euthanized for Limited dilution assay. (A) Lesion thickness in millimeters per days of infection; (B) Representative ear lesion photographs; (C) Quantification of parasite load per tissue gram of the ear; (D) and draining lymph node. The data (means ± standard deviations; n = 5) are representative of two independent experiments producing the same result profile. [file 13071_2025_7127_MOESM4_ESM.tif]

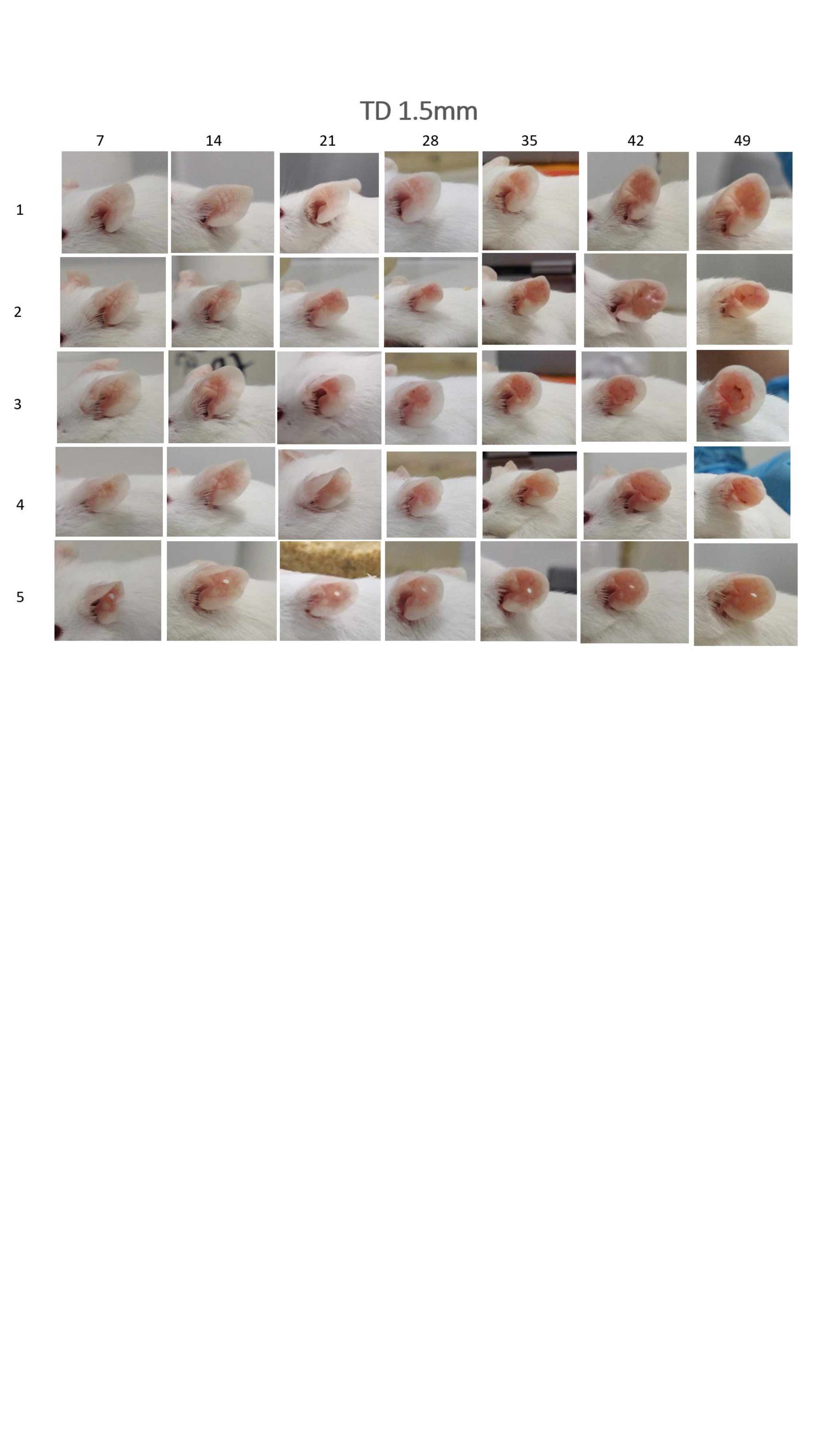

Supplement: Supplementary file 5 — Additional file 5: Figure S4. Configuration of 1.5-mm depth caused tissue destruction. Female BALB/c mice aged 6-8 weeks were infected with 2x10^6^ Leishmania amazonensis promastigotes in the stationary phase, either by transdermal route (TD) with different microneedle depth (1.5mm and 1.0mm). Weekly photographs of mice lesion macroscopic aspect was assessed over 49 days. The data (means ± standard deviations; n = 5) are representative of two independent experiments producing the same result profile. [file 13071_2025_7127_MOESM5_ESM.tif]
